# Aortic sodium [^18^F]fluoride uptake following endovascular aneurysm repair

**DOI:** 10.1136/heartjnl-2023-322514

**Published:** 2023-05-10

**Authors:** Samuel Debono, Jennifer Nash, Alexander J Fletcher, Maaz Syed, Edwin J R van Beek, Michelle Claire Williams, Orwa Falah, Andrew Tambyraja, Marc R Dweck, David E Newby, Rachael O Forsythe

**Affiliations:** 1 British Heart Foundation Centre for Cardiovascular Science, University of Edinburgh, Edinburgh, UK; 2 Department of Child Health, University of Glasgow, Glasgow, UK; 3 Edinburgh Imaging Facility, Queens Medical Research Institute, The University of Edinburgh, Edinburgh, UK; 4 The Edinburgh Vascular Service, Royal Infirmary of Edinburgh, NHS Lothian, Edinburgh, UK

**Keywords:** Aortic Aneurysm, Positron-Emission Tomography, Endovascular Procedures

## Abstract

**Objective:**

In patients with abdominal aortic aneurysms, sodium [^18^F]fluoride positron emission tomography identifies aortic microcalcification and disease activity. Increased uptake is associated with aneurysm expansion and adverse clinical events. The effect of endovascular aneurysm repair (EVAR) on aortic disease activity and sodium [^18^F]fluoride uptake is unknown. This study aimed to compare aortic sodium [^18^F]fluoride uptake before and after treatment with EVAR.

**Methods:**

In a preliminary proof-of-concept cohort study, preoperative and post-operative sodium [^18^F]fluoride positron emission tomography-computed tomography angiography was performed in patients with an infrarenal abdominal aortic aneurysm undergoing EVAR according to current guideline-directed size treatment thresholds. Regional aortic sodium [^18^F]fluoride uptake was assessed using aortic microcalcification activity (AMA): a summary measure of mean aortic sodium [^18^F]fluoride uptake.

**Results:**

Ten participants were recruited (76±6 years) with a mean aortic diameter of 57±2 mm at time of EVAR. Mean time from EVAR to repeat scan was 62±21 months. Prior to EVAR, there was higher abdominal aortic AMA when compared with the thoracic aorta (AMA 1.88 vs 1.2; p<0.001). Following EVAR, sodium [^18^F]fluoride uptake was markedly reduced in the suprarenal (ΔAMA 0.62, p=0.03), neck (ΔAMA 0.72, p=0.02) and body of the aneurysm (ΔAMA 0.69, p=0.02) while it remained unchanged in the thoracic aorta (ΔAMA 0.11, p=0.41).

**Conclusions:**

EVAR is associated with a reduction in AMA within the stented aortic segment. This suggests that EVAR can modify aortic disease activity and aortic sodium [^18^F]fluoride uptake is a promising non-invasive surrogate measure of aneurysm disease activity.

**WHAT IS ALREADY KNOWN ON THIS TOPIC**

- In abdominal aortic aneurysms, sodium [^18^F]fluoride identifies aortic microcalcification and is associated with faster aneurysm growth. Endovascular aneurysm repair durability is limited by the development of endoleaks and this necessitates the use of life-long postoperative surveillance programmes.

**WHAT THIS STUDY ADDS**

- This study demonstrates that endovascular aneurysm repair leads to a reduction in microcalcification activity in the abdominal aorta which can be detected as a reduction in aortic sodium [^18^F]fluoride uptake on positron emission tomography.

**HOW THIS STUDY MIGHT AFFECT RESEARCH, PRACTICE OR POLICY**

- This proof-of-concept study shows that this imaging technique can detect changes in microcalcification activity in abdominal aortic aneurysms. This technique holds promise as a non-invasive marker of disease activity and treatment efficacy in endovascular aneurysm repair.

## Introduction

Endovascular aneurysm repair (EVAR) has changed the elective treatment of abdominal aortic aneurysms. Compared with open surgical repair, it is a much less invasive procedure and is associated with lower perioperative morbidity and mortality and improved short-term outcomes.^1 2^ The stent graft provides a physical barrier that excludes the aneurysm from the circulation and reduces mortality from aneurysm rupture. EVAR use is limited by its durability, including the formation of endoleaks and stent migration necessitating life-long postoperative surveillance programmes.^3^ Following EVAR, shrinkage of the aneurysm sac can be observed, and this is taken as a positive morphological sign of procedural success.

Aortic aneurysm formation is characterised by medial wall atrophy and degeneration which may be triggered by an initial inflammatory response.^4^ This results in thinning, weakening and stiffening of the aortic wall which leaves it vulnerable to dilatation. These progressive inflammatory and degenerative disease processes trigger a vascular calcific response^5^ which is characterised by deposition of calcium-containing and phosphate-containing hydroxyapatite crystals and is termed microcalcification.^6^ This is distinct from the end-stage macrocalcification which can be readily identified by CT. Detection of the early microcalcific stages has the potential to identify ‘active’ vascular degeneration. Sodium [^18^F]fluoride is a positron emission tomography (PET) radiotracer which binds to the deposited hydroxyapatite crystals and can detect aortic microcalcification. In the Sodium [^18^F]Fluoride Imaging of Abdominal Aortic Aneurysms (SoFIA^3^ study (NCT02229006), aortic sodium [^18^F]fluoride uptake specifically localised to abdominal aortic aneurysms and was associated with more rapid aneurysm expansion. This was independent of age, sex, baseline diameter, body mass index, blood pressure, smoking, renal function or peripheral arterial disease. During follow-up, aneurysms with the highest sodium [^18^F]fluoride uptake expanded more rapidly and patients were most likely to require elective abdominal aortic aneurysm repair (because they reached the threshold of 55 mm) or to have experienced aneurysm rupture.^7^

Given that sodium [^18^F]fluoride PET-CT is a non-invasive marker of aortic aneurysm disease activity which predicts disease progression and clinical outcomes, we hypothesised that aortic sodium [^18^F]fluoride uptake would be reduced after successful EVAR implantation. In an exploratory proof-of-concept study, we undertook repeated sodium [^18^F]fluoride PET-CT in participants of the SoFIA^3^ study who had undergone EVAR.

## Methods

### Study population

The study population consisted of patients recruited into the Sodium [^18^F]Fluoride Imaging in Abdominal Aortic Aneurysms study (NCT02229006). Participants had an asymptomatic abdominal aortic aneurysm and were under ultrasound-based surveillance. Patients who had undergone successful elective EVAR were invited for recruitment into the PET-EVAR Study (Predicting Endoleaks Following Endovascular Aortic Aneurysm Repair Using Sodium [^18^F]Fluoride).

### Study design

This was a longitudinal observational study (NCT04577716) that was conducted with the written informed consent of all subjects, with approval by the South-East Scotland Research Ethics Committee (20/SS/0119), and in accordance with the Declaration of Helsinki. Study participants were studied at baseline and at a minimum of 1 year following EVAR.

### Study assessments

At each of the two study visits, participants underwent a clinical assessment prior to imaging. Patients were then administered a target dose of 125 MBq of sodium [^18^F]fluoride intravenously, and 60 min later they were imaged on a hybrid 128–slice PET-CT scanner (Biograph mCT, Siemens Healthineers, Erlangen, Germany). A low-dose attenuation correction CT scan was performed (120 kV, 50 mAs, 5/3 mm), followed by acquisition of PET data at 10 min intervals in three or four bed positions to cover the thoracic and abdominal aorta. A contrast-enhanced aortic CT angiogram (120 kV, 145 mAs, 3/3 mm, field of view 400 and 1/1 mm, field of view 300; triggered at 181 Hounsfield units) was performed on the same scanner immediately after PET acquisition. This was preceded by a non-contrast CT aortic calcium score at the second visit. Static PET-CT images were reconstructed with correction applied for attenuation, dead-time, scatter and random coincidences, using an optimised iterative reconstruction algorithm (ultra-High Definition; TrueX+Time-of-Flight, 2 iterations and 21 subsets, matrix 200, zoom 1; Gaussian filter 5 mm).

### Image analysis

#### Aortic morphology and calcium score

Aneurysm morphology, including preoperative and postoperative aortic sac size, neck length and length of common iliac arteries, was measured using Picture Archiving and Communications System (PACS, Carestream Health). Calcium score was measured using dedicated software (Vitrea Advanced, Toshiba Systems) and quantified as calcium score (Agatston units (AU)), calcium volume (mm^3^) and calcium mass (mg). The threshold for calcification was set at a computed tomographic density of 130 Hounsfield units having an area ≥1 mm^2^.^8^

#### Aortic microcalcification activity

**Figure 1:**
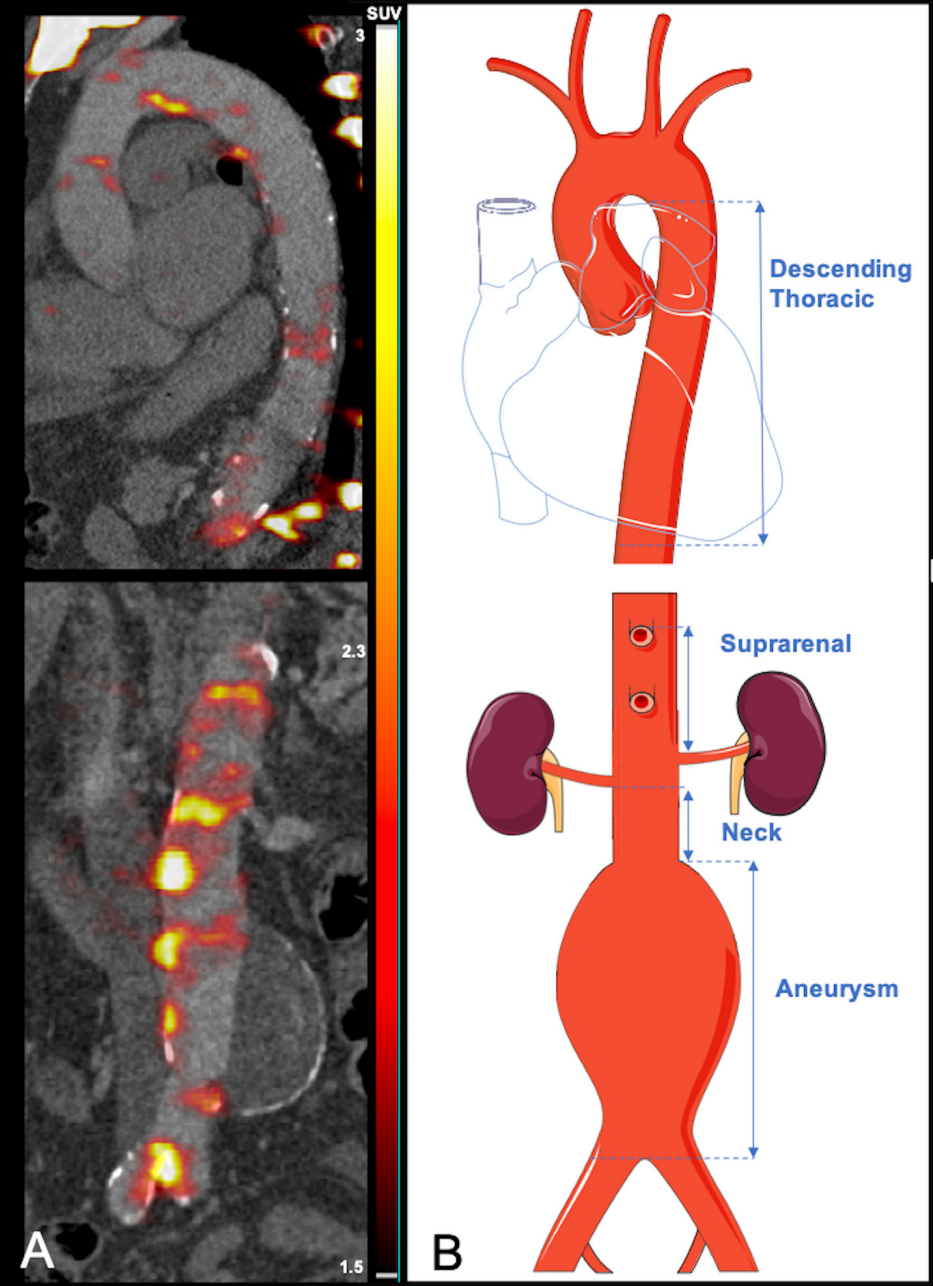
Regional analysis of aortic microcalcification activity. (A) Preoperative PET and CT with colour scale bar in SUVs. (B) Schematic representation demonstrating the analysed aortic regions (vertical arrows: descending thoracic, suprarenal, neck and aneurysm body). PET, positron emission tomography; SUVs, standard uptake values.

Sodium [^18^F]fluoride uptake was measured in four anatomically distinct regions of interest: the descending thoracic aorta, the suprarenal region, aneurysm neck and body of the aneurysm as described previously.^9^ The four regions were defined as: (i) the *descending thoracic aorta*—from the first trans-axial slice of the descending aorta to the aortic hiatus at the diaphragm; (ii) the *suprarenal* region—from the level of the origin of the coeliac artery to the upper-most renal artery (iii) the *neck*—from the lower-most renal artery to the aortic aneurysm and (iv) the *aneurysm body*—from the aneurysm neck to the aortic bifurcation (figure 1).

The aortic microcalcification activity (AMA), a summary measure of mean aortic sodium [^18^F]fluoride uptake in a 3D volume, was calculated for each region.^9^ In brief, AMA is calculated by measuring the mean radiotracer uptake value of each region using a custom validated tool (Fusion Quant v1.21.0421, Cedars-Sinai Medical Centre, Los Angeles), and then each region is normalised for its volume (cm^3^) and for the background blood pool activity of the right and left atria. The AMA analysis method also includes a threshold technique to correct for the spillover effect created by physiological radiotracer uptake in the vertebrae.^9^

### Statistical analysis

Continuous variables with normal distribution were presented as mean±SD and skewed continuous variables were presented as median (IQR). Categorical variables were presented as number (percentage). A simple main effect one-way model was used to assess regional difference in the AMA. A Kruskal-Wallis rank sum test was used to assess regional differences in the AMA between the timepoints. Wilcoxon signed-rank test was used to test preoperative and postoperative calcium scores. Statistical significance was taken as a two-sided p<0.05. All statistical analysis was performed in RStudio (V2022.02.3+492, RStudio, PBC).

## Results

Twelve of 72 participants of the original study population underwent elective EVAR. One participant was ineligible due to renal failure and one participant declined further participation. Ten patients were successfully recruited. They were predominantly male and had a mean age of 76±6 years (table 1). The mean interval between the preoperative and postoperative scans was 78±5 months and the time from EVAR to postoperative scan was 62±21 months.

**Table 1:** Patient characteristics at postoperative scan

Characteristic	N=10
Age (years)	76±6
Male	9 (90%)
Female	1 (10%)
Systolic blood pressure (mm Hg)	156±15
Diastolic blood pressure (mm Hg)	83±8
Heart rate (beats/min)	68±15
Body mass index (kg/m^2^)	27.1±3.2
*Medical history*	
Current or ex-smoker	10 (100%)
Hypertension	8 (80%)
Hypercholesterolemia	9 (90%)
Diabetes	2 (20%)
Ischaemic heart disease	4 (40%)
Peripheral arterial disease	2 (20%)
Cerebrovascular accident	3 (30%)
Chronic obstructive pulmonary disease	3 (30%)
No family history of aneurysm disease	10 (100%)
*Medications*	
Antiplatelet agents	7 (70%)
Anticoagulant agents	2 (20%)
Statins	9 (90%)
Beta-blockers	5 (50%)
Angiotensin-converting enzyme or angiotensin receptor blockers	4 (40%)
Calcium channel antagonist	3 (30%)

Mean±SD; number (%).

### Aortic morphology and calcium score

All treated aneurysms were infrarenal and had a mean aortic diameter of 57±2 mm at time of EVAR. Six patients had a concurrent iliac aneurysm. All stent grafts were inserted according to manufacturers’ instructions for use (table 2).

**Table 2:** Aortic morphology and stent graft details

	N=10^1^
Preoperative aortic size (mm)	57±2
Postoperative aortic size (mm)	51±12
Concurrent iliac aneurysm	6 (60%)
Preoperative iliac size (mm)	21±3
*Morphology*	
Length of neck (mm)	16 (15 to 26)
Neck angulation (°)	31 (29 to 44)
Left iliac length (mm)	54±24
Right iliac length (mm)	55±23
*Stent graft repair type*	
Cook	3 (30%)
Gore C3 Excluder	3 (30%)
Medtronic Endurant	4 (40%)

Mean±SD deviation; Median (IQR); number (%).

In eight patients (80%), there was no change, or there was a reduction in the aneurysm sac size following EVAR (mean change −6±12 mm). In the remaining two patients, there was an increase in sac size at follow-up. In one patient, there was a 14 mm increase due to an active type 2 endoleak. This was being actively managed and there had been multiple endovascular attempts to treat it (by embolisation and by CT-guided Onyx infiltration to the sac). In the second patient, there was a 6 mm increase. This patient had the shortest follow-up period of the cohort (13 months) and had previously demonstrated a type 2 endoleak at 8 months after EVAR.

The median postoperative thoracic calcium score was increased in all three measures when compared with the preoperative score (1924 vs 686 AU, p=0.02), but there were no differences in the suprarenal aorta (525 vs 232 AU, p=0.32) (table 3).

**Table 3:** Preoperative and postoperative aortic calcium scores

	Preoperative	Postoperative	P value
Thoracic calcium score, AU	686 (35 to 1528)	1924 (557 to 4698)	0.002
Thoracic calcium volume, mm^3^	718 (28 to 1323)	1638 (550 to 3838)	0.002
Thoracic calcium mass, mg	165 (8 to 383)	489 (132 to 1260)	0.002
Suprarenal calcium score, AU	232 (58 to 797)	524 (170 to 1138)	0.32
Suprarenal calcium volume, mm^3^	220 (58 to 764)	510 (211 to 926)	0.43
Suprarenal calcium mass, mg	58 (14 to 197)	129 (42 to 275)	0.19

Median (IQR).

AU, Agatston units.

### Aortic microcalcification activity

**Figure 2:**
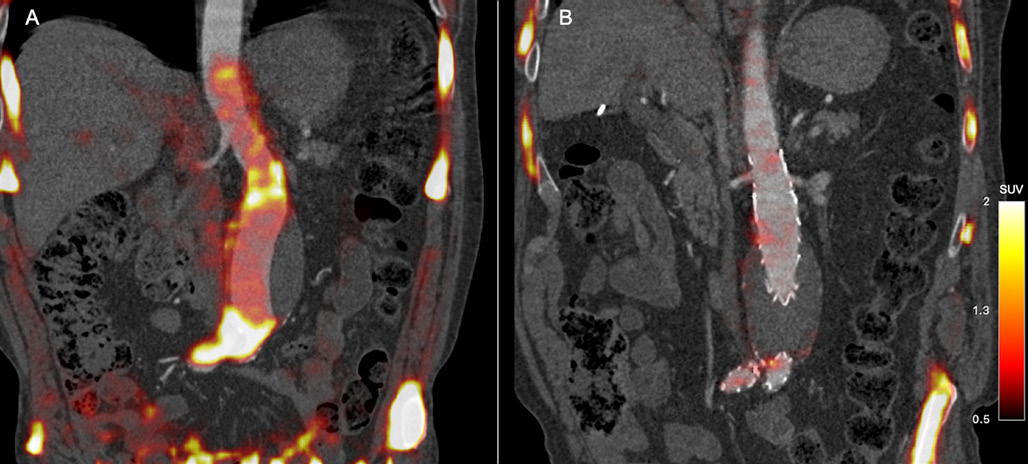
Images from a representative patient with a preoperative and a postoperative sodium [^18^F]fluoride positron emission tomography and CT angiogram. The CT angiogram is performed immediately after acquisition of PET data. A visual reduction in sodium [^18^F]fluoride uptake can be observed when comparing the preoperative PET-CT (Panel A) and the postoperative PET-CT (Panel B) in the same patient. SUVs represented in the colour scale bar. PET, positron emission tomography; SUVs, standard uptake values.

**Figure 3:**
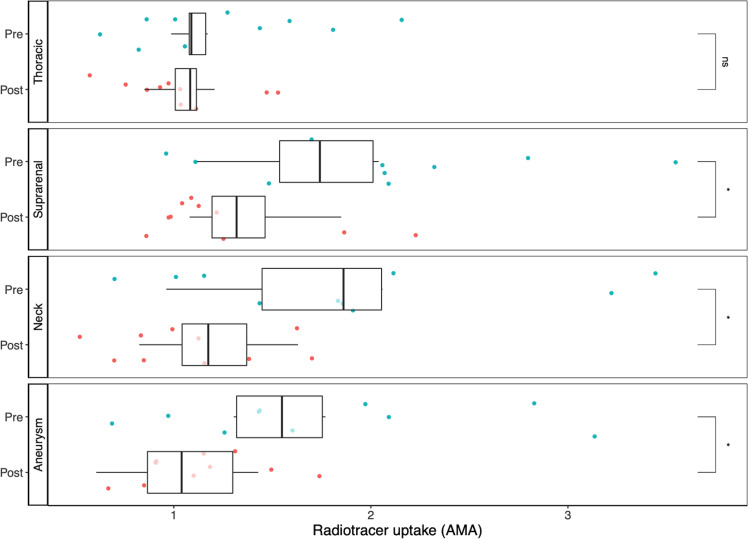
Regional aortic sodium [^18^F]fluoride uptake and aortic microcalcification activity in positron emission tomography and CT. Radiotracer uptake in the four aortic regions (descending thoracic, suprarenal, neck and aneurysm body) before (pre) after (post) endovascular aneurysm repair. AMA, aortic microcalcification activity. *p<0.05; ns, not significant.

The AMA at baseline was twice as high in all three abdominal aortic regions when compared with the thoracic aorta. Following EVAR, AMA was reduced in all abdominal aortic regions but not in the thoracic aorta (figure 2). The change in AMA was present in the suprarenal (0.62, p=0.03), neck (0.72, p=0.02) and aneurysm body (0.69, p=0.02) but not in the thoracic aorta (0.11, p=0.41; figure 3, table 4).

**Table 4:** Preoperative and postoperative aortic microcalcification activity

Aortic region	Preoperative mean (min–max)	Postoperative mean (min–max)	Change in AMA	P value
Thoracic	1.20 (0.99–1.69)	1.09 (0.85–1.47)	−0.11	0.41
Suprarenal	1.98 (1.12–3.6)	1.36 (1.08–1.85)	−0.62	0.03
Neck	1.93 (0.96–3.54)	1.21 (0.82–1.63)	−0.72	0.02
Aneurysm body	1.74 (0.6–3.24)	1.05 (0.61–1.43)	−0.69	0.02

Mean AMA values across each aortic region, and change in AMA following EVAR.

AMA, aortic microcalcification activity; EVAR, endovascular aneurysm repair.

A positive correlation was observed between the change in thoracic calcium score and the preoperative AMA (R=0.33, p=0.04). However, no relationships were observed between the preoperative, postoperative and change in AMA, and the aortic sac size or the change in aortic sac size.

The patient with an active endoleak during the postoperative visit had the highest AMA in the suprarenal and neck regions and the second highest AMA in the thoracic and aneurysm region on the preoperative PET-CT. Despite also demonstrating a reduction, this patient’s postoperative AMA was the highest of the cohort in all four aortic regions.

## Discussion

EVAR is associated with shrinkage of the abdominal aortic aneurysm sac: a key morphological indicator of procedural success.^10^ For the first time, we have shown that EVAR is also associated with a reduction in sodium [^18^F]fluoride uptake within the abdominal aorta. This reduction was prominent in the regions of the abdominal aorta covered by the stent graft and was not apparent in the thoracic aorta. This suggests that EVAR protects the aortic aneurysm from rupture by providing a physical barrier and by reducing aortic aneurysm disease activity within the aortic wall itself.

Calcification is a marker of disease burden and disease progression in coronary artery disease. Coronary sodium [^18^F]fluoride uptake correlates with progression of coronary calcification^11 12^ and is a marker of coronary atherosclerotic disease activity.^13–15^ It is also associated with ischaemic stroke^16 17^ and the future risk of myocardial infarction.^18^ The significance of aortic macrocalcification and the associated aortic Agatston calcium score is not well documented for aortic aneurysm disease. In a retrospective study of patients undergoing CT angiography as part of elective treatment work-up of abdominal aortic aneurysm disease, Chowdhury and colleagues observed that higher aortic calcium scores are associated with poorer outcomes.^19^ Conversely, in subthreshold abdominal aortic aneurysms, Klopf and colleagues observed an inverse relationship between increased abdominal aortic calcification and aneurysm disease progression over a 6-month period.^20^ Here, we observed an association between the change in thoracic calcium score and the preoperative AMA suggesting that a generally higher aortic disease activity correlates with increasing macrocalcification within the aorta. This underlines the relationship between vascular sodium [^18^F]fluoride uptake and aneurysm disease activity.

An increase in aneurysm diameter and a change in radiotracer uptake over time has previously been observed using 2-[^18^F]fluoro-2-deoxyglucose, where patients whose aneurysm diameter increased, had demonstrated lower radiotracer uptake on baseline PET-CT 9 months previously.^21^ This observation was attributed to cyclical periods of inflammation within the aneurysm wall and periods of aneurysm expansion. Rather than inflammation, sodium [^18^F]fluoride, detects microcalcification, which is increasingly becoming a specific and reliable marker of disease activity within the aorta. Sodium [^18^F]fluoride uptake quantifies microcalcification within the aortic media of patients with thoracic aortopathy^22^ and is associated with greater aortic growth after acute aortic syndrome.^23^ In the present study, AMA was highest in the baseline PET-CT of the abdominal aorta where there is active abdominal aortic aneurysm disease. It is also noteworthy that one of the highest sodium [^18^F]fluoride uptakes in the cohort occurred in a patient with a type 2 endoleak and sac enlargement. Although the small cohort size is insufficient to draw definitive conclusions, it does provide promising preliminary data to suggest that high aortic sodium [^18^F]fluoride uptake may reflect disease activity. As an indicator of reduced aneurysm metabolic activity, suppression of uptake could confirm or predict treatment efficacy of EVAR and may have a role in a select group of patients who demonstrate sac expansion on routine screening.

The reduction in AMA we observed following EVAR suggests insertion of the stent graft can decrease aneurysm disease activity. The mechanism of such reduced disease activity has not been addressed by our study, but this could plausibly include reducing biomechanical stress within the aortic wall, preventing the build-up of further luminal thrombus or inflammatory mediators or providing a physical barrier to luminal blood pressure which could otherwise exacerbate aneurysmal dilatation. In turn, these processes could lead to a reduction or cessation in further degeneration in the aortic media which leads to reduced deposition of hydroxyapatite crystals and a reduction in surface area for sodium [^18^F]fluoride binding. Irrespective of the mechanism, our findings highlight the potential of PET to provide an imaging biomarker of treatment efficacy in abdominal aortic aneurysm disease, especially for EVAR but potentially also for other future treatment interventions. Conversely, failure to suppress aortic sodium [^18^F]fluoride uptake may herald the development of complications and endoleaks not yet visible on CT. Speculatively, it could guide baseline patient selection for EVAR if intense uptake at baseline heralds likely future treatment failure, and is the subject of an ongoing prospective study (NCT04577716).

This is a small single centre proof-of-concept study, with the small sample size a result of recruitment of only those patients from the original SoFIA^3^ study who underwent EVAR. As such, our findings are preliminary and require further external validation. We should also acknowledge several other limitations of this pilot study. First, scan analysis was performed without blinding to the timing of the scans and clearly the aortic stent graft is readily visible on the attenuation correction CT. Second, although the AMA method used to quantify sodium [^18^F]fluoride uptake has shown excellent levels of agreement with conventional PET quantification methods,^9^ it has not been externally validated. Third, survival bias may play a role, although the majority (83%) of patients who underwent an EVAR operation from the original cohort were recruited into the study. Fourth, since the patients recruited in the study underwent their baseline scan at different timepoints in the natural history of their disease, their baseline radiotracer uptake, time to EVAR and follow-up time differed across the cohort. However, this does not preclude a comparison of uptake before and after EVAR. Fifth, the PET reconstruction algorithm uses the attenuation correction CT which is not specifically designed to account for the presence of a stent graft. However, in coronary studies, the presence of a stent usually results in overestimation of the PET signal rather than a reduction in signal.^24^ Finally, it was not possible to calculate the calcium score for all abdominal aortic regions due to the presence of the stent graft.

In conclusion, aortic sodium [^18^F]fluoride uptake localises to the diseased regions of abdominal aortic aneurysm disease and predicts development of aortic macrocalcification. Successful EVAR is associated with a marked reduction in aortic sodium [^18^F]fluoride uptake and microcalcification activity which is most prominent in the stented regions of the aorta. Although it requires future prospective validation and evaluation, this technique holds promise as a marker of aneurysm disease activity and treatment efficacy and may have clinical implications in personalised EVAR surveillance.

## Data Availability

Data are available on reasonable request.
